# Artificial intelligence−enhanced electrocardiography for hypertension: Prediction of incident hypertension and risk stratification

**DOI:** 10.1001/jamacardio.2024.4796

**Published:** 2025-03-01

**Authors:** Arunashis Sau, Joseph Barker, Libor Pastika, Ewa Sieliwonczyk, Konstantinos Patlatzoglou, Kathryn A. McGurk, Nicholas S. Peters, Declan P. O’Regan, James S. Ware, Daniel B. Kramer, Jonathan W. Waks, Fu Siong Ng

**Affiliations:** 1 National Heart and Lung Institute, Imperial College London, UK; 2 Department of Cardiology, Imperial College Healthcare NHS Trust, London, UK; 3 MRC Laboratory of Medical Sciences, Imperial College London, London, UK; 4 University of Antwerp and Antwerp University Hospital, Antwerp, Belgium; 5 Cardiovascular Research Center, Massachusetts General Hospital, Boston, MA, USA; 6 Department of Cardiology, Royal Brompton & Harefield Hospitals, Guy’s and St. Thomas’ NHS Foundation Trust, London, UK; 7 Richard A. & Susan F. Smith Center for Outcomes Research in Cardiology, Beth Israel Deaconess Medical Center, Harvard Medical School, Boston MA USA; 8 Harvard-Thorndike Electrophysiology Institute, Beth Israel Deaconess Medical Center, Harvard Medical School, Boston, MA, USA; 9 Department of Cardiology, Chelsea and Westminster Hospital NHS Foundation Trust, London, UK

## Abstract

**Importance:**

Hypertension underpins significant global morbidity and mortality. Early lifestyle intervention and treatment is effective in reducing adverse outcomes. Artificial intelligence-enhanced electrocardiography (AI-ECG) has been shown to identify a broad spectrum of subclinical disease, and may be useful for predicting incident hypertension.

**Objective:**

To develop an AI-ECG Risk Estimator (AIRE) to predict incident hypertension and stratify risk for hypertension associated adverse outcomes.

**Design:**

Development and external validation prognostic cohort study.

**Setting:**

Model development at Beth Israel Deaconess Medical Center (BIDMC), Boston USA, a secondary care setting. External validation in the UK Biobank (UKB), a UK based volunteer cohort.

**Participants:**

ECG algorithm trained on 1,163,401 ECGs from 189,539 patients.at BIDMC. 19,423 BIDMC patients comprising the test set, were evaluated for incident hypertension. UKB, 65,610 ECGs from same number of participants, 35,806 were evaluated for incident hypertension.

**Main Outcomes and Measures:**

The AI-ECG Risk Estimator for Hypertension (AIRE-HTN), which uses a residual convolutional neural network architecture with a discrete-time survival loss function, was trained to predict incident hypertension.

**Results:**

AIRE-HTN predicted incident hypertension (BIDMC: n = 6446 (33%) events, C-index 0.70 (0.69-0.71); UKB: n = 1532 (4%) events, C-index 0.70 (0.69-0.71).

Performance was maintained in subjects without LVH and those with normal ECGs (C-indices 0.67-0.72). AIRE-HTN was significantly additive to existing clinical risk factors in predicting incident hypertension (continuous net reclassification index 0.439 (0.332-0.530) BIDMC; 0.317 (0.233-0.373 UKB)).

In adjusted Cox models, AIRE-HTN score was an independent predictor of cardiovascular death (Hazard Ratio (HR) per standard deviation; 2.24 (1.67-3.00), heart failure HR 2.60 (2.22-3.04), myocardial infarction HR 3.13 (2.55-3.83), ischaemic stroke HR 1.23 (1.11-1.37), and chronic kidney disease HR 1.89 (1.68-2.12), beyond traditional risk factors.

**Conclusions and Relevance:**

We have developed AIRE-HTN, an AI-ECG model for predicting incident hypertension and identifying patients at risk of hypertension-related adverse events, beyond conventional clinical risk factors.

## Introduction

Hypertension affects 1 in 3 adults worldwide and accounts for approximately 20% of all US deaths (1,2). As a primary risk factor for cardiovascular disease, hypertension underpins a significant portion of the global disease burden (3).

Hypertension mediated organ damage is an early indicator of inadequate blood pressure control, with multinational guidelines advocating more aggressive treatment strategies when detected (4). Left ventricular hypertrophy (LVH) is a form of hypertension mediated cardiovascular damage and is an independent predictor for extracardiac damage (5). LVH has been detectable on electrocardiograms (ECGs) since the early 20th century (6). However ECG LVH criteria have limited diagnostic utility, requiring further imaging to screen for hypertension mediated cardiac damage (4).

Artificial intelligence-enhanced electrocardiography (AI-ECG) offers the potential to detect subtle ECG changes, including features not appreciable to humans, offering insights beyond traditional ECG interpretation (7). A major advantage of AI approaches is the ability to extract features relevant to the specific task, without anchoring on prior beliefs (8,9).

We recently described the AI-ECG Risk Estimation platform (AIRE), capable of predicting mortality, future atherosclerotic cardiovascular disease, ventricular arrhythmia and heart failure (8). In this study we expand AIRE to identify subjects at risk of incident hypertension, and stratify the risk of hypertension-related sequelae. We additionally explore the biological plausibility of the AI-ECG tool and perform explainability analyses in support of clinical application.

## Methods

### Ethical approvals

This study complies with all relevant ethical regulations, further details are provided in the Supplementary eMethods.

### Cohorts

We studied two cohorts. The Beth Israel Deaconess Medical Center (BIDMC), Boston, USA a secondary care cohort consisting of routinely collected health record data and the UK Biobank (UKB) cohort, a longitudinal study of volunteers between 40 and 69 years of age upon joining during 2006 to 2010. A subset of these UKB individuals, who lived near an imaging assessment centre, were invited for the second follow up visit and had an ECG performed. More information about these cohorts and blood pressure measurements can be found in the Supplementary eMethods. This study has been reported in accordance with the TRIPOD statement.

### Model development

We expanded the AIRE platform for prediction of incident hypertension (AIRE-HTN). Hypertension included both essential and secondary hypertension types. Hypertension and outcomes were defined using ICD-9/10 diagnostic codes in the BIDMC dataset and algorithmically defined diseases (a combination of ICD codes, self-reported diagnoses, primary care records and causes of death) in UKB.

We used a two-stage process to develop AIRE-HTN, both stages use an end-to-end neural network. ECG pre-processing and dataset splits (eFigure 1) are described in the Supplementary eMethods.

We first developed an AI-ECG hypertension classification model using the BIDMC cohort as the derivation dataset. The model architecture was based on convolutional neural networks that incorporates residual blocks, with a final sigmoid layer (10). The model was trained to identify prevalent hypertension as a classification task.

Next, to predict incident hypertension, AIRE-HTN was further developed by adapting the final layer to accommodate a discrete-time survival loss function (11). This approach allows AIRE-HTN to account for both time to outcome (incident hypertension) and censorship (i.e., loss to follow up). The classification model described above was used for the weight initialisation of the AIRE-HTN model. The outputs of the AIRE-HTN model are termed the AIRE-HTN score, which was standardised and normalised. Higher values indicate higher risk of incident hypertension. The model output at 5 years was used for prediction of incident hypertension (irrespective of the duration of follow up for that particular subject). All ECGs were used for model training, however, as prediction of incident hypertension is most relevant for outpatients, only outpatient ECGs were used in the BIDMC test set. Further details of hyperparameters and model training are in the Supplementary eMethods.

### Explainability analysis

To explore the ECG morphologies and standard ECG metrics linked to the AIRE-HTN score, we used three approaches.

First, a variational autoencoder (VAE) was trained on median ECG beats. Median beats were derived using the BRAVEHEART ECG analysis software (12). The latent features derived from the VAE were then fed into a linear regression model, aiming to predict the output of AIRE-HTN, a continuous range between 0-1 that relates to incident hypertension risk. The VAE was used only for explainability analysis and not to create the AIRE-HTN model (which used an end-to-end neural network as described above). We identified and visualised the top three most significant features, based on their t-values, through a process known as latent feature traversal. Further details available in Supplementary eMethods.

Second, we calculated the median waveform from the 1,000 ECGs with the lowest and highest AIRE-HTN score to qualitatively explore the morphologies associated with risk.

Third, we performed univariable correlation between AIRE-HTN and ECG parameters. Using linear regression, AIRE-HTN was adjusted for age, age^2^, height, weight, body surface area (BSA) and waist circumference.

### Left ventricular hypertrophy definitions

Left ventricular hypertrophy (LVH) was defined in accordance with American Society of Echocardiography guidelines (13), as an anteroseptal or inferolateral wall thickness of greater than or equal to 0.9cm in females and 1.0cm in males. LVH ECG criteria were defined using the Sokolow-Lyon criteria, where the S wave in V1 plus the R wave in V5 or V6 were added, and if the sum was greater than 35 mm, LVH was present (14). A continuous sum of maximum voltages for S waves in V1/V2 and R waves in V5/V6 was also calculated. Echocardiographic LV mass was calculated using the linear method as previously described (13). Cardiac MRI (CMR) is the gold standard for identification of LVH (15). Therefore, in the UKB, CMR was used to identify subjects with normal LV mass based on previously described age and sex specific normal ranges (16). Normal ECG definition is described in the Supplementary eMethods.

### Survival and statistical analyses

AIRE-HTN score quartiles were defined using the distributions in the validation set for Kaplan-Meier curves. AIRE-HTN score quartiles were plotted, and statistical significance assessed using the log rank test. For incident hypertension analyses, subjects with hypertension diagnosis at baseline (both cohorts) or within 30 days after the ECG (in BIDMC) were excluded. Subjects without follow up data (i.e. they were censored on the day of the ECG) were also excluded. Cox model analyses were used to predict outcomes from the first ECG per subject only. The AIRE-HTN score variable was standardised, so hazard ratios reflect one standard deviation changes in AIRE-HTN score. Cox models were fit using the test dataset for BIDMC and UKB datasets. For prediction of incident hypertension, clinical covariates were age, sex, systolic blood pressure (SBP), diastolic blood pressure (DBP), smoking status, prevalent diabetes mellitus (DM) and ethnicity. For UKB analyses body mass index (BMI) was additionally included. For prediction of adverse events, covariates were age, sex, SBP, DBP, smoking status, diabetes mellitus, hypertension, hyperlipidaemia and ethnicity. UKB analyses additionally included BMI and number of anti-hypertensives as covariates. Due to the smaller number of patients with BMI data available, BMI was not included in primary BIDMC analyses, but is included in sensitivity analyses. Medication data were not available in the BIDMC cohort. Subjects were censored at the time of death, last in-person hospital contact (BIDMC) or the UKB national censoring dates. To measure the improvement in prediction performance gained by AIRE-HTN, continuous net-reclassification index (NRI) was calculated. Causal mediation analysis was performed using the R package mediation (17). We calculated the proportion mediated using logistic regression for 5 year incident outcomes, adjusted for smoking status, diabetes mellitus, hyperlipidaemia and ethnicity. We investigated the proportion mediated by a diagnosis of hypertension, which could be at baseline or during follow up (but prior to the adverse event). Statistical analyses were performed with R 4.2.0 statistical package (R Core Team, Vienna, Austria) or Python (version 3.9).

### Phenome-wide association study

We performed phenome wide association studies (PheWAS) to better understand the biology underlying AIRE-HTN scores. We used the UK Biobank cohort that contains data from over 3000 phenotypes derived from patient measurements, surveys, and investigations. We also applied this approach to the subjects with echocardiograms within 60 days of the ECG in the BIDMC test set. Using linear regression, the residual of AIRE-HTN score was calculated, after adjusting for age, age^2^, sex, height, weight and BSA. In the UK Biobank, waist circumference was additionally included as a covariate; these data was not available for the BIDMC cohort. Univariate correlation was then performed using the residual to investigate the association between AIRE-HTN scores and phenotypes or cardiac imaging parameters (cardiac magnetic resonance (CMR) and echocardiography). Further methods are described in the Supplementary eMethods.

## Results

### AI-ECG can predict incident hypertension

**Figure 1:**
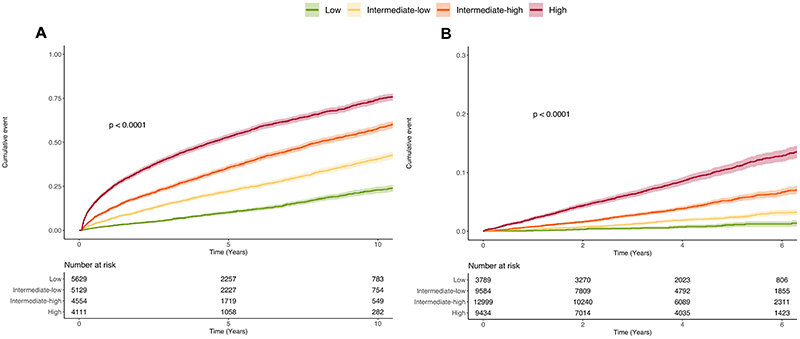
AIRE-HTN predicts incident hypertension AIRE-HTN stratified risk of incident hypertension in the BIDMC (A) and UKB (B) cohorts. Kaplan-Meier curves show cumulative probabilities of hypertension for the four quartiles of risk defined by AIRE-HTN predictions using a single ECG.

In the BIDMC cohort, 1,163,401 ECGs were available from 189,539 subjects, 47.9% were male. A total of 34,938 (18.4%) subjects died during follow-up (eTable 1). We used the first outpatient ECG in subjects without hypertension for evaluation of incident hypertension prediction (n = 19423, mean follow up 6.8 years±5.6 years, 6446 (33.2%) events). AIRE-HTN predicted incident hypertension with C-index 0.701 (0.69-0.71) with risk quartiles displayed in Figure 1 and eTable 2. The high-risk quartile had a four-fold greater risk of incident hypertension after adjustment for age and sex (HR 4.02 (3.65-4.43), p<0.0001). Performance was maintained in males and females and across major ethnic groups (Supplementary eFigure 2, eTable 3). In the BIDMC cohort we performed sensitivity analysis in subjects with normal wall thickness on echocardiography (C-index 0.69 (0.67-0.72)), absence of LVH by ECG criteria (C-index 0.70 (0.69-0.71), cardiologist-reported normal ECGs (C-index 0.72 (0.68-0.75) and normal baseline BP (<120/80 mmHg, C-index 0.73 (0.72-0.74)). Performance was maintained in the external validation UKB cohort (C-index 0.70 (0.69-0.71), (n = 35806, mean follow up 4.0±1.6 years, 1532 (4.3%) events). UKB sensitivity analysis was performed in subjects with normal LV mass by CMR (C-index 0.70 (0.69-0.72) and normal baseline BP (<120/80 mmHg) with no antihypertensive medications (C-index 0.71 (0.61-0.80).

In the UKB we found the AIRE-HTN score was associated with the number of medications prescribed at follow up (eFigure 3). In a subset of subjects with protocolised follow up and no diagnosis of hypertension or prescription of antihypertensives at baseline, AIRE-HTN predicted future anti-hypertensive prescription at the time of follow up (C-index: 0.71 (0.64-0.77). We additionally found AIRE-HTN was superior to continuous measures of LVH (including ECG voltage criteria and LV mass) for the prediction of incident hypertension (Supplementary Results).

**Figure 2:**
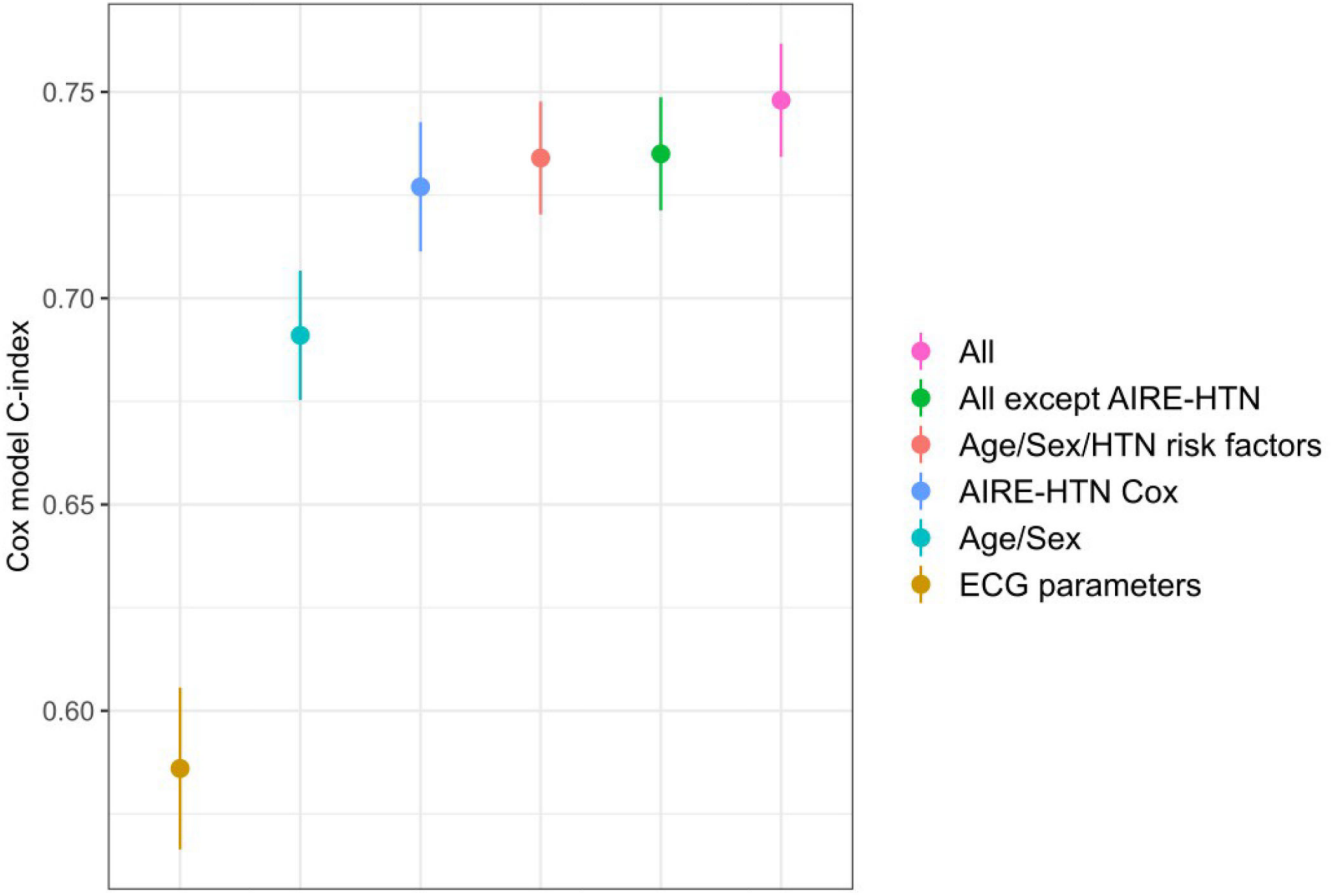
Cox models for prediction of incident hypertension Hypertension prediction C-index results comparing AIRE-HTN with clinical risk prediction methods for the prediction of incident hypertension. AIRE-HTN-Cox includes AIRE-HTN, age, sex and ECG parameters. HTN risk factors include: systolic blood pressure (SBP), diastolic blood pressure (DBP), smoking status, prevalent diabetes mellitus (DM) and ethnicity

We then compared AIRE-HTN score with existing clinical characteristics associated with hypertension; age, sex, systolic blood pressure (SBP), diastolic blood pressure (DBP), smoking status, prevalent diabetes mellitus (DM) and ethnicity. For UKB analyses body mass index (BMI) was additionally included. We found AIRE-HTN was significantly additive to existing clinical markers in predicting incident hypertension: (Figure 2 and eFigure 4, eTable 4), C-index with: 0.75 (0.73-0.76) vs without: 0.73 (0.72-0.75) AIRE-HTN, p < 0.0001). AIRE-HTN score provided additive predictive value as measured by the continuous NRI (BIDMC: 0.44 (0.33-0.53), UKB 0.32 (0.23-0.37)).

### AIRE-HTN predicts adverse events

**Figure 3:**
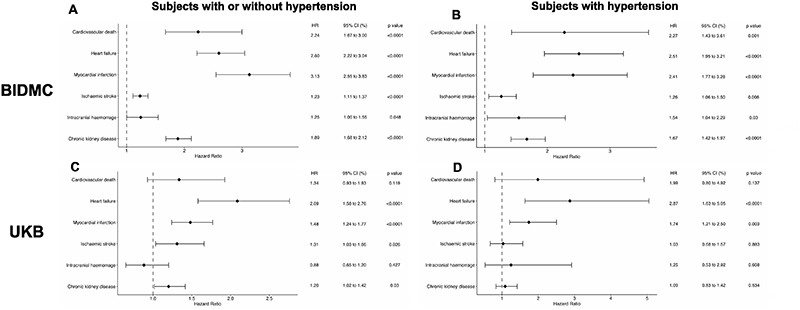
AIRE-HTN score is independently associated with hypertension related adverse outcomes In adjusted Cox models, AIRE-HTN score is an independent predictor of hypertension related adverse outcomes in subjects without existing cardiovascular/renal disease (**A + C**) and without existing cardiovascular/renal disease but with hypertension (**B + D**). Covariates for BIDMC analysis: age, sex, SBP, DBP, smoking status, prevalent DM, prevalent hypertension, prevalent hyperlipidaemia and ethnicity. UKB analyses additionally included BMI and number of anti-hypertensives as covariates. Hazard ratio refers to one standard deviation increase of AIRE-HTN score.

We assessed the association of the AIRE-HTN score with hypertension related adverse outcomes, after adjusting for clinical covariates (listed in Methods). We found that the AIRE-HTN score was an independent predictor of cardiovascular death (Hazard Ratio (HR) per standard deviation (SD); 2.24 (1.67-3.00), heart failure HR 2.60 (2.22-3.04), myocardial infarction HR 3.13 (2.55-3.83), ischaemic stroke HR 1.23 (1.11-1.37), and chronic kidney disease HR 1.89 (1.68-2.12), p values all <0.0001) in BIDMC outpatients. These findings were broadly consistent regardless of hypertension status and across cohorts (Figure 3, eFigure 5, eTable 5-6).

We subsequently explored if AIRE-HTN score was associated with adverse outcomes mediated through a diagnosis of hypertension. We found AIRE-HTN score was associated with cardiovascular death with partial mediation through a diagnosis of hypertension (proportion mediated 11% (4-30%, p <0.0001) to 35% (17%-167%, p < 0.0001) depending on cohort and subgroup, eTable 7). We found varying degrees of mediation for the other adverse events, from no-significant mediation for haemorrhagic stroke to 57% for CKD (31-110%, p < 0.0001).

### Explainable ECG morphologies are associated with AIRE-HTN scores

**Figure 4:**
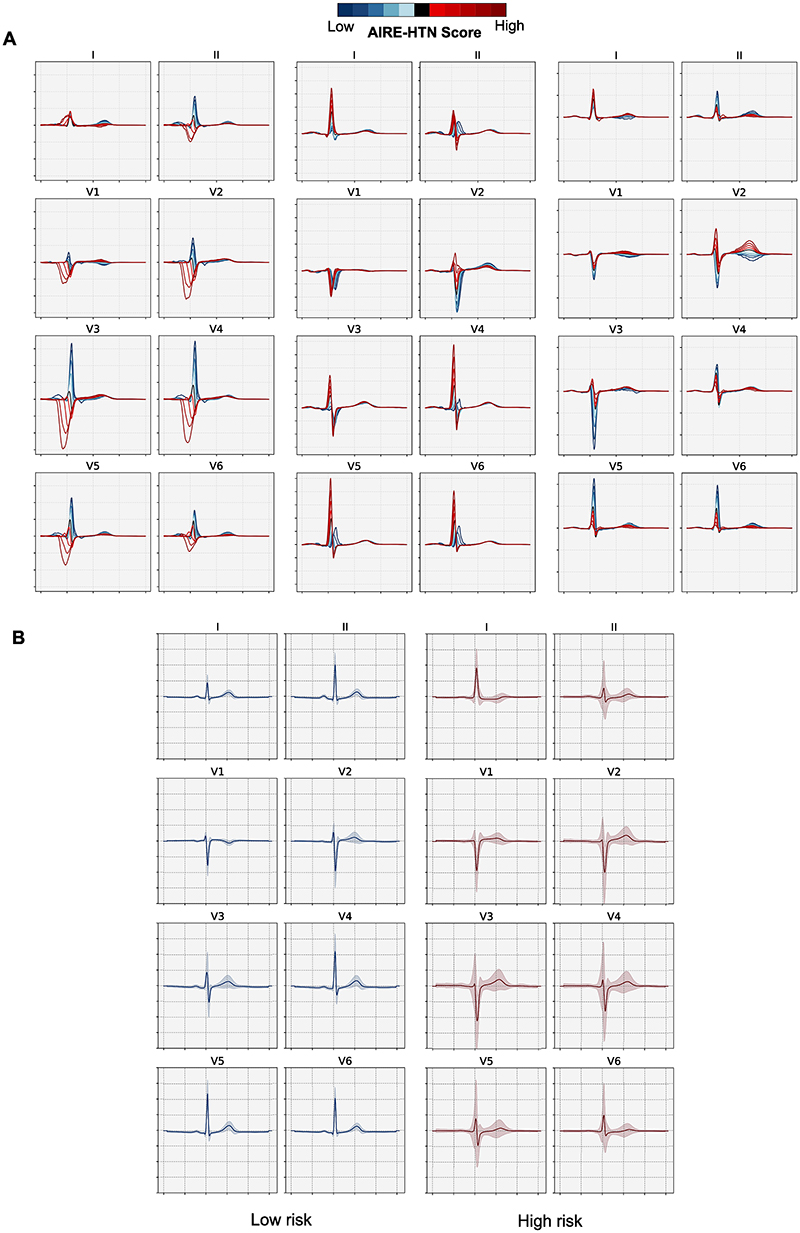
Explainability analyses A) A variational auto-encoder was used to identify the most important morphological features in AIRE-HTN score, each subpanel shows one of three latent features. (B) Average ± standard deviation (shaded region) ECG waveforms for the 10000 highest and lowest AIRE-HTN score from the BIDMC test set.

To explore the ECG morphological changes responsible for the AIRE-HTN score, we used a VAE to visualise the features most associated with the AIRE-HTN score. Figure 4A shows the three latent features most highly associated with AIRE-HTN scores (t values = 325, 177 and 174 respectively), including intraventricular conduction delay, QRS amplitude and T wave variation in lead II as most important variation between high and low risk ECGs.

Next using median beats, we plotted the average representations of the 10000 ECGs with the highest and lowest AIRE-HTN scores (Figure 4B). We found delayed R to S transition from V3 in low-risk groups to V5 in high-risk groups, combined with much greater heterogeneity in QRS complex and T waves in high-risk groups.

Finally, we assessed the correlation of established ECG parameters with AIRE-HTN scores (eFigure 6). These three methods highlight QRS voltage (R = 0.22, p < 0.0001), QRS duration (R = 0.05, p < 0.0001),, R wave progression and axis (R = 0.13, p < 0.0001),, PR interval (R = 0.15, p < 0.0001),, T wave morphology and QT interval (R = 0.08, p < 0.0001), as important factors in the derivation of AIRE-HTN scores.

### Biological exploration: Phenotypic associations of AIRE-HTN score

**Figure 5:**
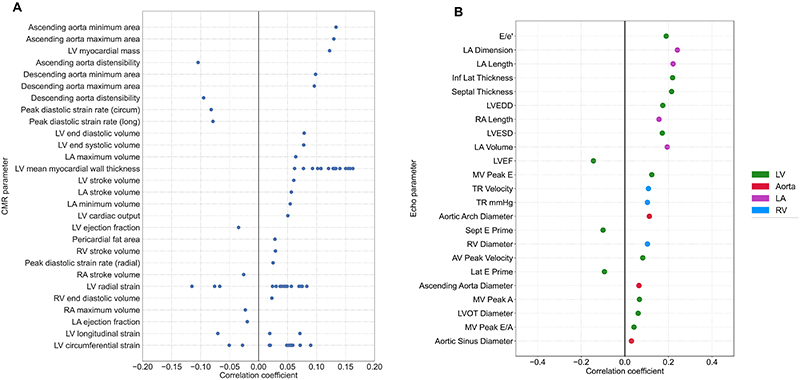
Cardiac imaging associations of AIRE-HTN score Univariate correlation between AIRE-HTN score and cardiac MRI, UKB cohort (**A**) and echocardiographic, BIDMC cohort (**B**) parameters was performed. Variables with multiple points indicate results of multiple measurements at varying anatomical locations. Comparisons meeting significance after Bonferroni correction are shown. MRI: magnetic resonance imaging, LA: left atrium, RA: right atrium, LV: left ventricle, RV: right ventricle.

To investigate the biological associations with AIRE-HTN score and support model credibility, we performed a PheWAS in the UK Biobank. First we examined the correlations of AIRE-HTN score with age, sex, SBP and DBP and in particular found a modest correlation with age (R = 0.40, p < 0.001) (Supplementary eResults). We then correlated AIRE-HTN score with over 3000 biological and clinical variables in the UKB, with covariates including age and age squared. We investigated associations with echocardiographic parameters in the BIDMC cohort. A Manhattan plot depicts the significant associations in UKB (eFigure 7). In particular, echocardiographic and CMR parameters included independent associations with LV wall thickness and mass (R = 0.16, p < 0.0001), measures of diastolic function and filling pressure (R = 0.19, p < 0.0001) and aortic dimensions (R = 0.13, p < 0.0001) (Figure 5). Other significant positive correlations included carotid intima-media thickness (R = 0.07, p < 0.0001), measured blood pressure (R = 0.21, p < 0.0001), arterial stiffness (R = 0.06, p < 0.0001) and measures of adiposity (R = 0.07, p < 0.0001). Significant negative correlations included peak heart rate (R = 0.07, p < 0.0001) on exercise and birthweight (R = 0.07, p < 0.0001).

## Discussion

In this paper, we introduce AIRE-HTN, a biologically credible AI model for ECG-based prediction of incident hypertension and risk stratification for adverse events.

The AI-ECG signature for hypertension has been previously explored, focusing on estimating blood pressure from ECG morphology (18), diagnosing LVH (19) and prevalent hypertension (20–23). Unlike these existing AI-ECG hypertension studies, AIRE-HTN leverages large, unselected cohorts, is externally validated, and focuses on prediction of incident hypertension and risk stratification. AIRE-HTN retains its predictive accuracy in the absence of electrical or structural LVH and in cardiologist reported “normal” ECGs.

The ability of AIRE-HTN to predict incident hypertension was additive to traditional markers for both cohorts, whilst previous statistical models have shown LVH as the only ECG criterion predictive for incident hypertension (24). Importantly, AIRE-HTN is an independent predictor of cardiovascular death, heart failure, myocardial infarction, ischaemic stroke and chronic kidney disease in both populations. Using this model to predict incident hypertension and its link to cardiovascular outcomes could help identify at risk patients for whom more active surveillance and lifestyle interventions (25–28) might be recommended at an earlier stage in the life course.

Although the current approach to hypertension treatment relies on population derived thresholds of blood pressure measurement, alternative biomarkers such as AIRE-HTN risk scores might increase awareness of the continuous pathogenic vascular syndrome that is labelled hypertension. The consistent model performance in a group of subjects with a normal baseline BP and no LVH suggests AIRE-HTN may be identifying the latent biology of hypertension pathogenesis, and not just simply identifying subjects with milder hypertension at baseline

Our explainability and biological plausibility analyses, designed to support the credibility of AIRE-HTN, highlight VAE ECG features associated with higher AIRE-HTN scores and are consistent with the literature for human derived features of hypertension including LVH ECG criteria (29), resting heart rate (30), ECG strain (31), QRS duration and QT interval (32). Importantly, correlations with ECG LVH criteria were relatively weak, demonstrating that AIRE-HTN predictions are not dependent on them. Associations with CMR and echocardiographic parameters align with expectations typical of hypertensive remodelling; increased left ventricular mass, larger aortic dimensions, diminished LV strain, lower ejection fractions and impaired diastolic function.

PheWAS identified biologically plausible associations, reinforcing the reliability of AIRE-HTN. Associations included established hypertension correlates, such as measures of adiposity, smoking status and increased carotid intimal thickness (33). Negative associations with AIRE-HTN risk scores included birthweight (34), and peak heart rate during exercise.

### Limitations

The inherent limitations to epidemiological studies include the accuracy of coded outcomes within the clinical datasets. Hypertension within BIDMC was defined using ICD codes, which lack granularity and may not match contemporary guidelines. We were unable to validate our findings against the gold standard of ambulatory monitoring. However, sensitivity analysis with baseline blood pressure recordings and medication use, and external validation with algorithmically defined diseases, demonstrated consistent findings. While AIRE-HTN has been internally and externally validated in a large number of subjects, its performance across diverse populations and clinical settings remains to be explored. The incident hypertension model shows only modest discriminative capability; however, it was significantly additive to traditional risk stratification methods and superior to commonly used clinical risk tools in other clinical scenarios (35). The UKB PheWAS results were drawn from a population of European ancestry and require validation in other populations. The UKB cohort is known to consist of a relatively healthy population (36) and may not reflect the general population, in contrast BIDMC is a relatively unhealthy hospital-based population. Importantly, AIRE-HTN was able to discriminate risk of hypertension in both cohorts.

## Conclusion

We have developed AIRE-HTN, an AI-ECG model for prediction of incident hypertension and for risk stratification for hypertension associated adverse events. AIRE-HTN is significantly additive to known clinical predictors of hypertension. Through exploratory and phenotypic analyses, we have demonstrated the biological plausibility of these findings. Enhanced predictability could influence surveillance programs and primordial prevention.

## Supplementary Material

Supplement

## Data Availability

UK Biobank data are available upon application (http://www.ukbiobank.ac.uk/). The BIDMC dataset is restricted due to ethical limitations. The programming code relating to these analyses is available upon request to the corresponding author.

## Key points

### Question

Can an ECG-based artificial intelligence risk estimator for hypertension (AIRE-HTN) predict incident hypertension and stratify risk for incident hypertension associated adverse events?

### Findings

AIRE-HTN predicts incident hypertension (C-index (0.70) and stratifies risk for cardiovascular death HR 2.24 (1.67-3.00), heart failure HR 2.60 (2.22-3.04), myocardial infarction HR 3.13 (2.55-3.83), ischaemic stroke HR 1.23 (1.11-1.37) and chronic kidney disease HR 1.89 (1.68-2.12).

### Meaning

AIRE-HTN can predict the development of hypertension and may identify at-risk patients for enhanced surveillance.
