# Supplementary material for: Artificial intelligence−enhanced electrocardiography for hypertension: Prediction of incident hypertension and risk stratification
Source: JAMA Cardiol. Author manuscript; Available in PMC 2025 Mar 26. (PMC11904724; doi:10.1001/jamacardio.2024.4796)
Supplement: Supplement [file EMS203668-supplement-Supplement.pdf]

Artificial intelligence–enhanced electrocardiography for hypertension: Prediction of incident hypertension and risk stratification

Supplementary materials

Supplementary eMethods

Ethical approvals

The Beth Israel Deaconess Medical Center (BIDMC) cohort ethics review and approval was provided by the Beth Israel Deaconess Medical Center Committee on Clinical Investigations, IRB protocol # 2023P000042, waiver of consent was granted. The UK Biobank (UKB) has approval from the North West Multi-Centre Research Ethics Committee as a Research Tissue Bank (application IDs 48666, 40616 and 47602) informed consent was obtained by all participants.

ECG datasets

A priori sample size calculations were not performed. Missing data was handled by complete-case analysis. Ethnicity was participant reported.

(i) The BIDMC cohort

The BIDMC cohort is a dataset comprised of routinely collected data from Beth Israel Deaconess Medical Center, Boston, USA. Subject over 16 years old with a valid ECG performed from 2014 to 2023 were included. Prior ECGs back to 2000 were included for these subjects. Mortality was determined via the Massachusetts Department of Public Health (DPH) and/or review of the BIDMC electronic medical record, while diagnostic International Classification of Diseases (ICD) codes were used to determine disease status. Subjects were censored at time of death or last in-person hospital contact.

(ii) The UK Biobank Cohort

The UKB is longitudinal study of over 500,000 volunteers aged 40-69 at the time of enrolment in 2006-2010 (1). At baseline assessment participants provided information on health and lifestyle via questionnaire, had physical measures taken (including height, weight, and blood pressure) and donated samples of blood urine and saliva. A subgroup of participants were invited back for subsequent visits for additional investigations, including for detailed studies including cardiac magnetic resonance imaging (MRI), brain MRI and digital ECGs. There is evidence of healthy volunteer selection bias (2). Outcomes were linked to cancer and death registry data, hospital admissions and primary care records. Detailed phenotyping using the cardiac MRI data has been previously described (3, 4). Importantly, the UK Biobank was a prospective, rigorously conducted, volunteer cohort study. ECG recordings were protocolised and performed consistently across patients and sites. As the subjects were volunteers, the ECGs were performed solely for the cohort study and not for any clinical reason. Therefore, there is also no confounding based on the timing of the ECG in relation to any clinical care or diagnoses.

ECG pre-processing

The 12-lead ECG data was first assessed for quality using the BRAVEHEART software and signals with excessive artefact excluded (5). Next pre-processing was performed, which included applying a bandpass filter from 0.5 to 100Hz, a 60Hz notch filter, and resampling at 400Hz. To accommodate a 10-second recording, zero padding was used, resulting in a total of 4096 samples for each lead. Considering that leads III, aVL, aVR, and aVF are derived from leads I and II and do not offer unique data, they were excluded from the model development and evaluation.

Consequently, the input dimension for a single ECG to the neural network model was 4096 x 8.

Dataset splits
The dataset was divided into training, tuning, and internal testing segments following a 50/10/40% split, respectively (eFigure 1). The splits were executed based on patient ID and stratified according to the availability of ECGs with corresponding 5-year life status information. ECGs from single individuals were exclusively allocated to one of the training, validation, or testing groups such that a person with multiple ECGs would only be represented in one split.

Model training
The output of the model is a predicted probability of survival free from hypertension within each discrete time-interval. The model was trained to account for events occurring 10 years from the time of the ECG. The model was selected based on the lowest validation loss and was evaluated on the unseen test set. Hyperparameter optimisation was performed using the BIDMC validation set. Models were trained for up to 50 epochs and the lowest validation loss of each training run used to evaluate model performance and select optimal hyperparameters. The hyperparameters tuned were the learning rate, batch size and discrete-time survival timepoints. Remaining hyperparameters were used as previously described without further tuning (6). Models were trained using a single Nvidia RTX 6000 on Imperial College London's high performance computing cluster. The Keras framework with a TensorFlow backend was used for neural network training and inference (7, 8).

Variational auto-encoder training

We trained a variational autoencoder (VAE) as previously described (9) using median ECG beats. The VAE consists of three components: the encoder, the decoder and the latent space. The encoder and the decoder are made up of one-dimensional convolutional layers with increasing filters and decreasing kernel sizes closer to the latent space. The latent space was restricted to 30 features at a maximum, although typically only a subset of these features was used by the model for the reconstruction. The model was trained to minimize both the median ECG reconstruction loss, defined by a symmetric mean absolute percentage error function, and the Kullback-Leibler divergence (KL loss). This second term is specifically added to the VAE model to ensure that the features generated by the model are generative and disentangled. An additional β-parameter was included as a weight on the KL-term to optimize the balance between the reconstruction loss and the latent factor interpretability. We tested beta values of 0.1, 0.25, 0.5, 1, 3, 5 and 10 and defined the best model at a β-parameter of 0.25 based on the Pearson correlation between the median and its reconstruction in the validation dataset, as well as a visual inspection of the latent vector transversals.

Latent factor visualizations represent traversals of that factor within a range of -5 to 5 (step of 1), while keeping all other factors at zero. This allows the independent visualisation of the morphological changes identified by each latent factor. By visualising the most important latent factors, we can interpret the morphological features more important for AIRE-HTN predictions

Cox models proportional hazards
Recent work suggests virtually all real-world clinical datasets will violate the proportional hazards assumptions if sufficiently powered and that statistical tests for the proportional hazards assumption may be unnecessary (10). In line with these recommendations, the proportional hazards assumption was not evaluated and the hazard ratio from our Cox models should be interpreted as a weighted average of the true hazard ratios over the follow-up period.

Normal ECG definition
To explore if the model was useful in the evaluation of “normal” ECGs we explored a subset of the BIDMC dataset that had cardiologist reports. We determined “normal” by searching for ‘normal ecg’ in the free text reports - a whole word match was required to exclude ‘abnormal ecg’ – and the phrase ‘otherwise’ was excluded. Additionally, we filtered by heart rate (60-100 bpm), PR interval (less than 200ms), QRS duration (less than 120ms) and QTc interval (less than 470ms). Clinician ECG reports were not available in the UK Biobank.

Baseline blood pressure
In the real-world BIDMC dataset, SBP and DBP readings were taken as per routine clinical practice. We used the average of all available readings 180 days before and after the ECG. While in the UKB where protocolised BP readings were taken, we used the readings on the same day as the ECG. In the UKB blood pressure was measured using an automated Omron blood pressure monitor. An appropriate size cuff is placed on the participant’s arm. The participant was asked to place their arm on the desk top, so that the cuff was at about same level as their heart, and to breathe in and out slowly five times in a relaxed fashion. The rubber inflation tubing is connected to the Omron blood pressure monitor and the reading was taken. Normal baseline BP was taken as less than 120/80 in accordance with AHA/ACC guidelines (11).

Supplementary eResults

AIRE-HTN correlations with clinical covariates:
We found AIRE-HTN had modest correlations with age ($r = 0.40$, $p < 0.001$), male sex ($r = 0.20$, $p < 0.001$), SBP ($r = 0.37$, $p < 0.001$) and DBP ($r = 0.23$, $p < 0.001$).

AIRE-HTN compared to continuous measures of LVH:

We calculated the sum of maximum voltages for S waves in V1/V2 and R waves in V5/V6. This had a C-index of 0.495 (0.485-0.506) for the prediction of incident hypertension (AIRE-HTN C-index 0.701 (0.694-0.708).

To further investigate the validity of AIRE-HTN in comparison to LVH for prediction of incident hypertension, we additionally investigated the predictive value of LV mass as calculated by echocardiography (BIDMC: C-index 0.630 (0.613-0.646) vs AIRE-HTN: 0.700 (0.685-0.714), $p < 0.0001$) and cardiac MRI (UKB: 0.629 (0.612-0.645) vs AIRE-HTN: 0.701 (0.686-0.716), $p < 0.0001$).

eFigure 1
Schematic showing dataset splits for model development and validation. BIDMC: Beth Israel Deaconess Medical Center.

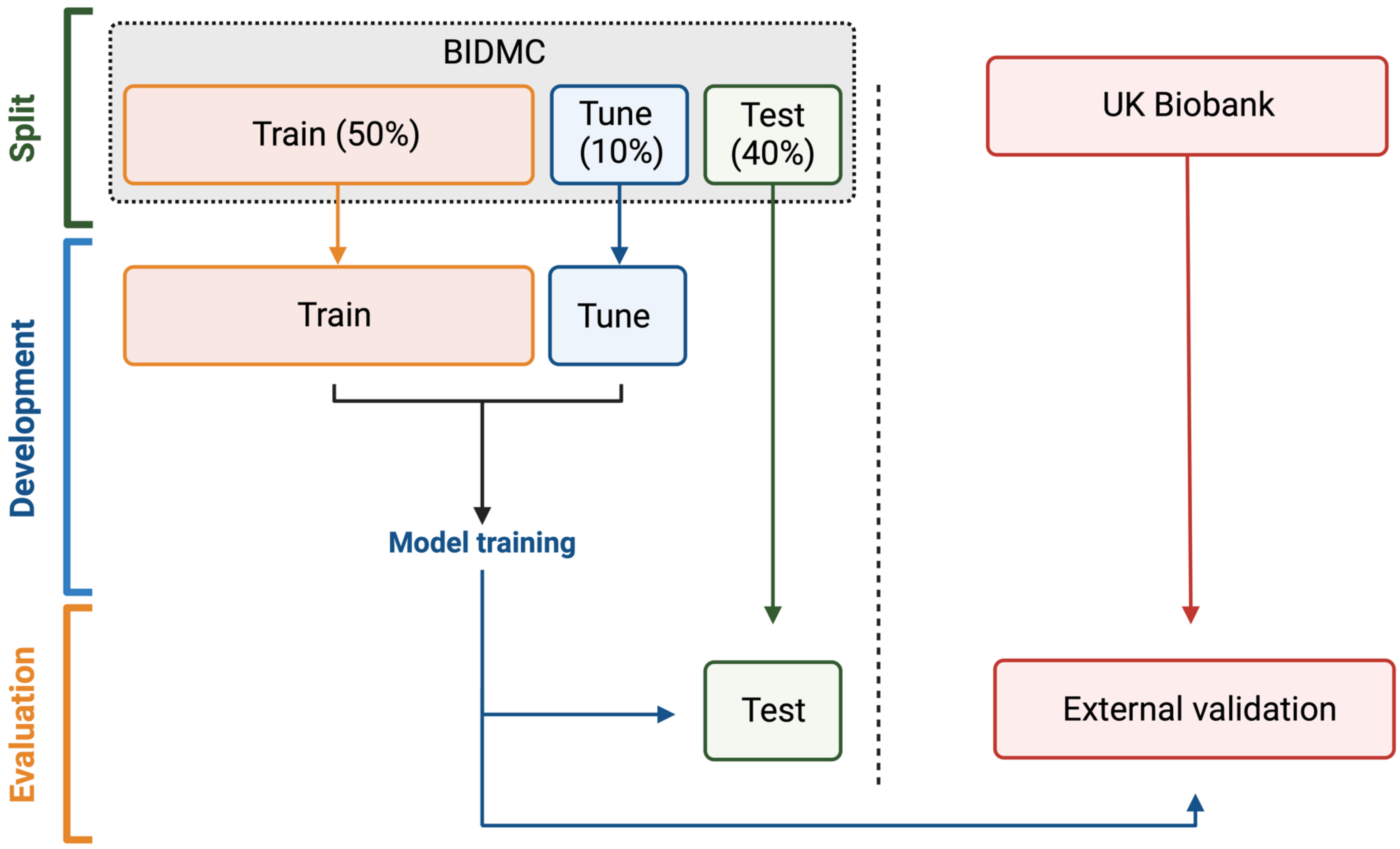

eFigure 2: AIRE-HTN, incident hypertension prediction performance stratified by gender and ethnicity

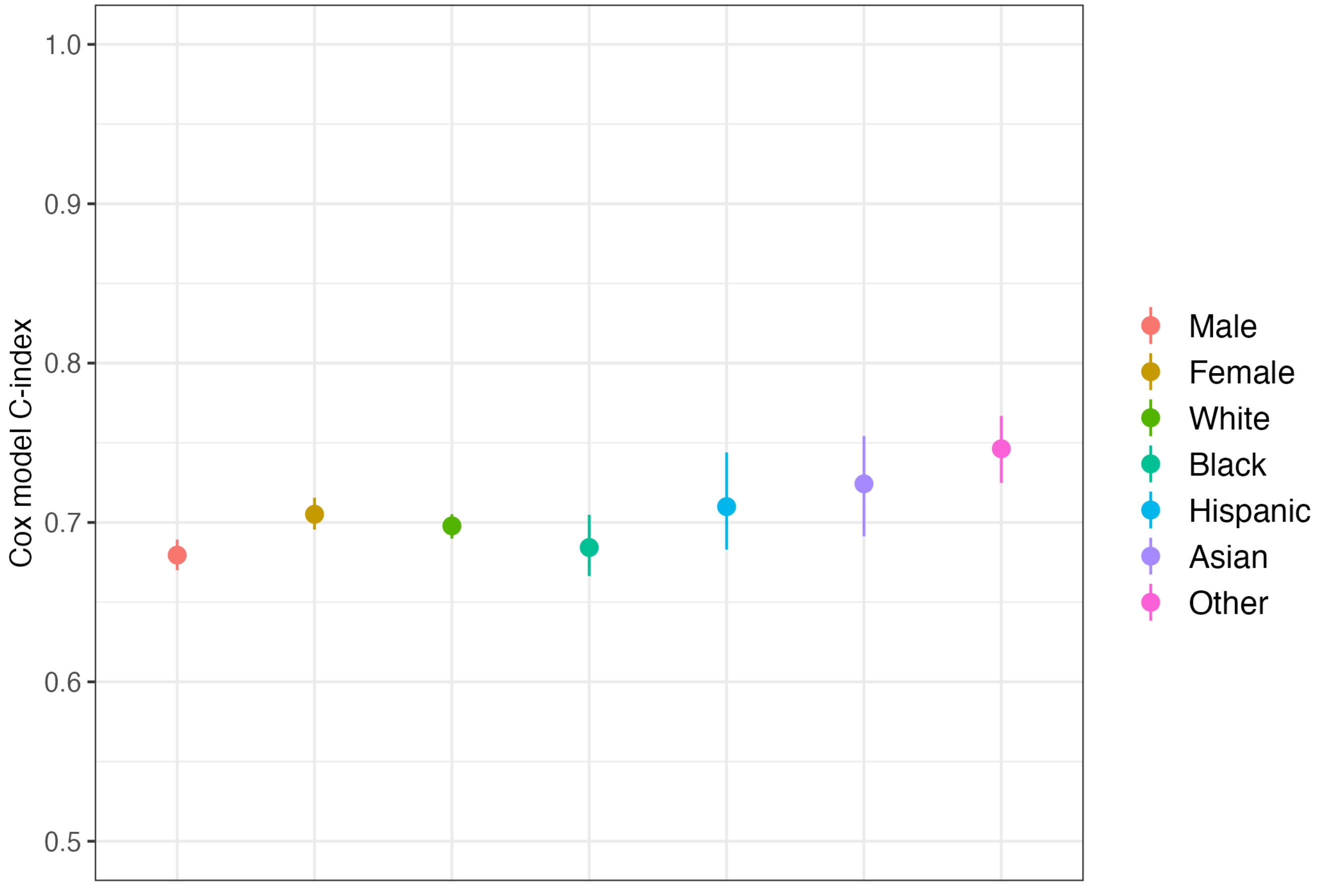

eFigure 3
Violin plot showing the association between AIRE-HTN score at baseline and the number of prescribed antihypertensives at the follow up visit in the UKB cohort.

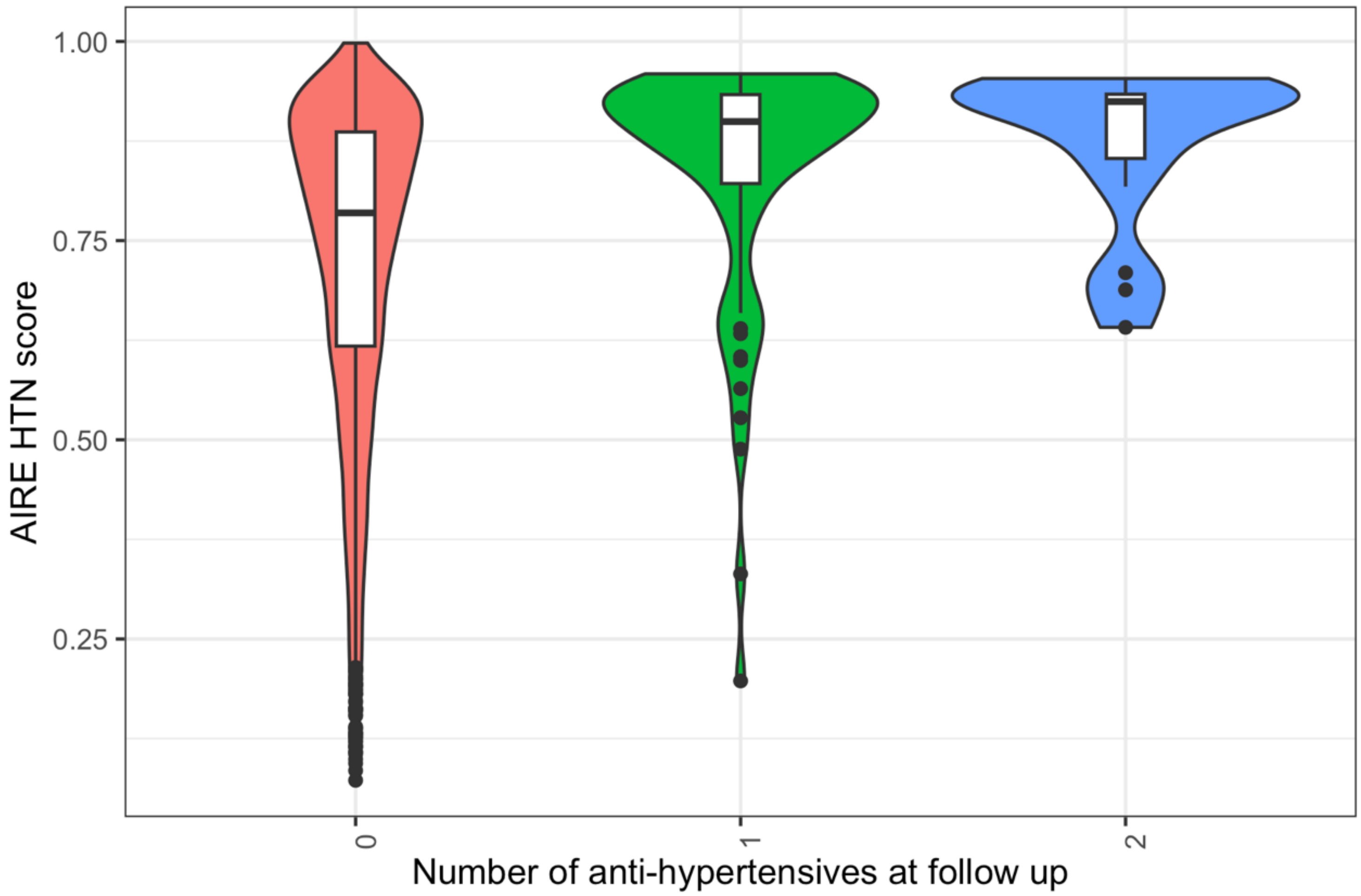

eFigure 4

Cox models for prediction of incident hypertension, including blood results

Hypertension prediction C-index results comparing AIRE-HTN with clinical risk prediction methods for the prediction of incident hypertension. AIRE-HTN-Cox includes AIRE-HTN, age, sex and ECG parameters.

HTN risk factors include: systolic blood pressure (SBP), diastolic blood pressure (DBP), smoking status, prevalent diabetes mellitus (DM) and ethnicity. Bloods include serum sodium, potassium and creatinine taken in the 30 days prior to the ECG.

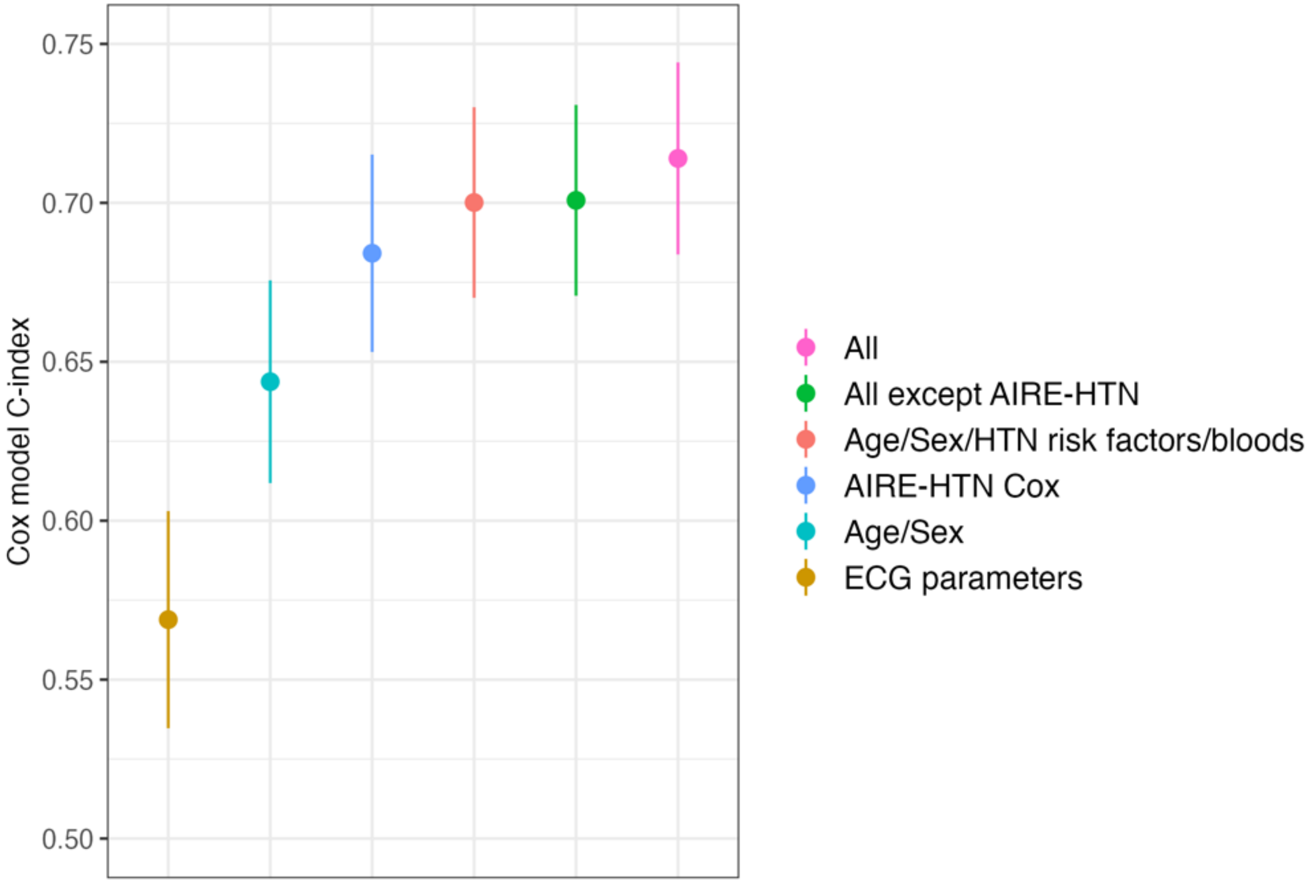

eFigure 5

Sensitivity analysis for Figure 2 now including BMI as a covariate in BIDMC cohort. Covariates: age, sex, SBP, DBP, smoking status, prevalent DM, prevalent hypertension, prevalent hyperlipidaemia, BMI and ethnicity

In adjusted Cox models, AIRE-HTN score is an independent predictor of hypertension related adverse outcomes in subjects without existing cardiovascular/renal disease (**A**) and without existing cardiovascular/renal disease but with hypertension (**B**).

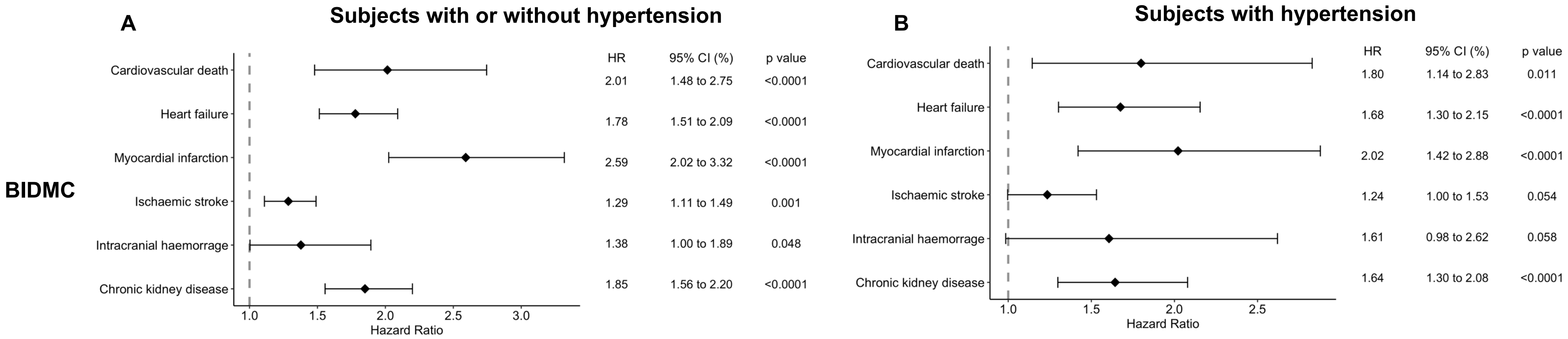

eFigure 6
AIRE-HTN score correlations with ECG parameters
Univariate correlation was performed to evaluate the association of AIRE-HTN score with common ECG parameters. P value for all correlations < 0.001

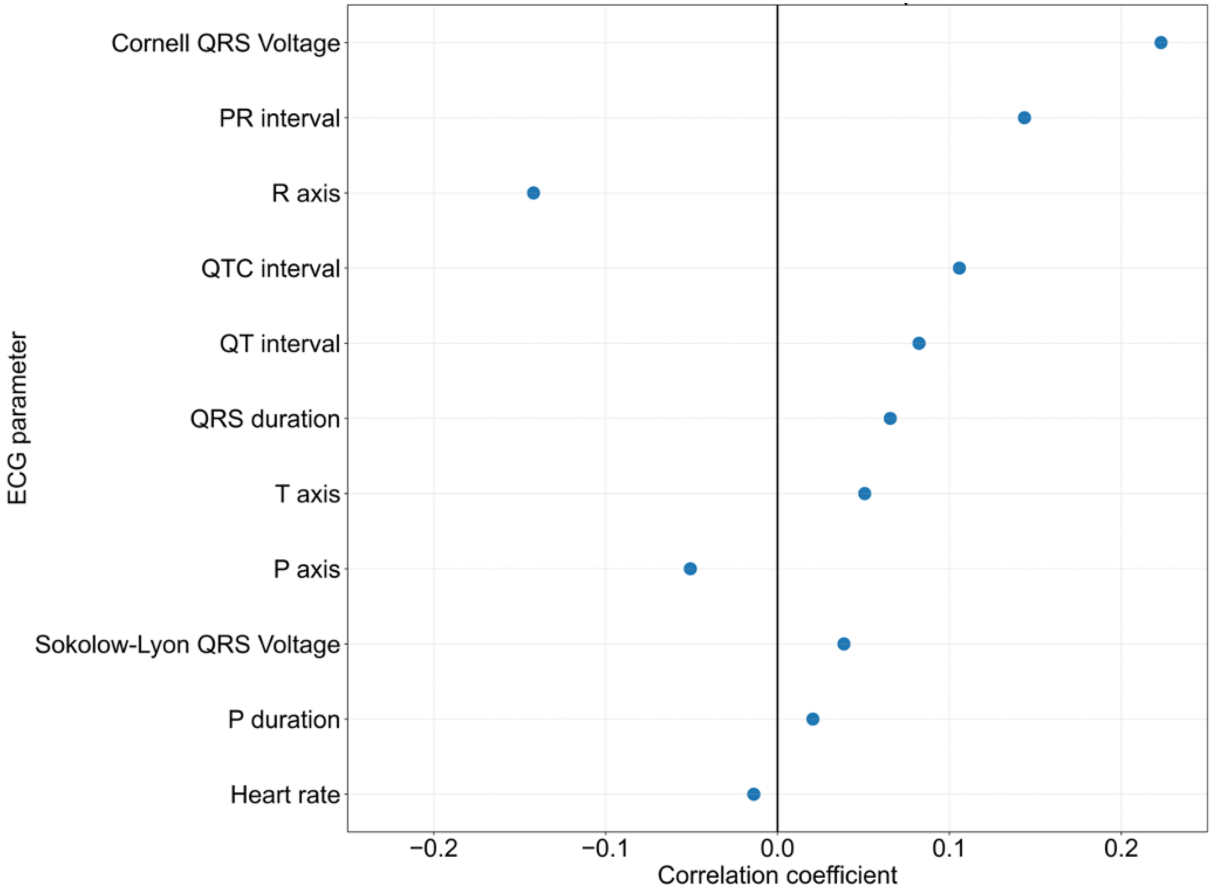

eFigure 7: UK Biobank Phenome-wide association study (PheWAS). Manhattan plot showing the univariate correlation coefficient between AIRE-HTN and UK Biobank parameters. Large dots indicate associations crossing the threshold for statistical significance after Bonferroni correction, small dots are not statistically significant.

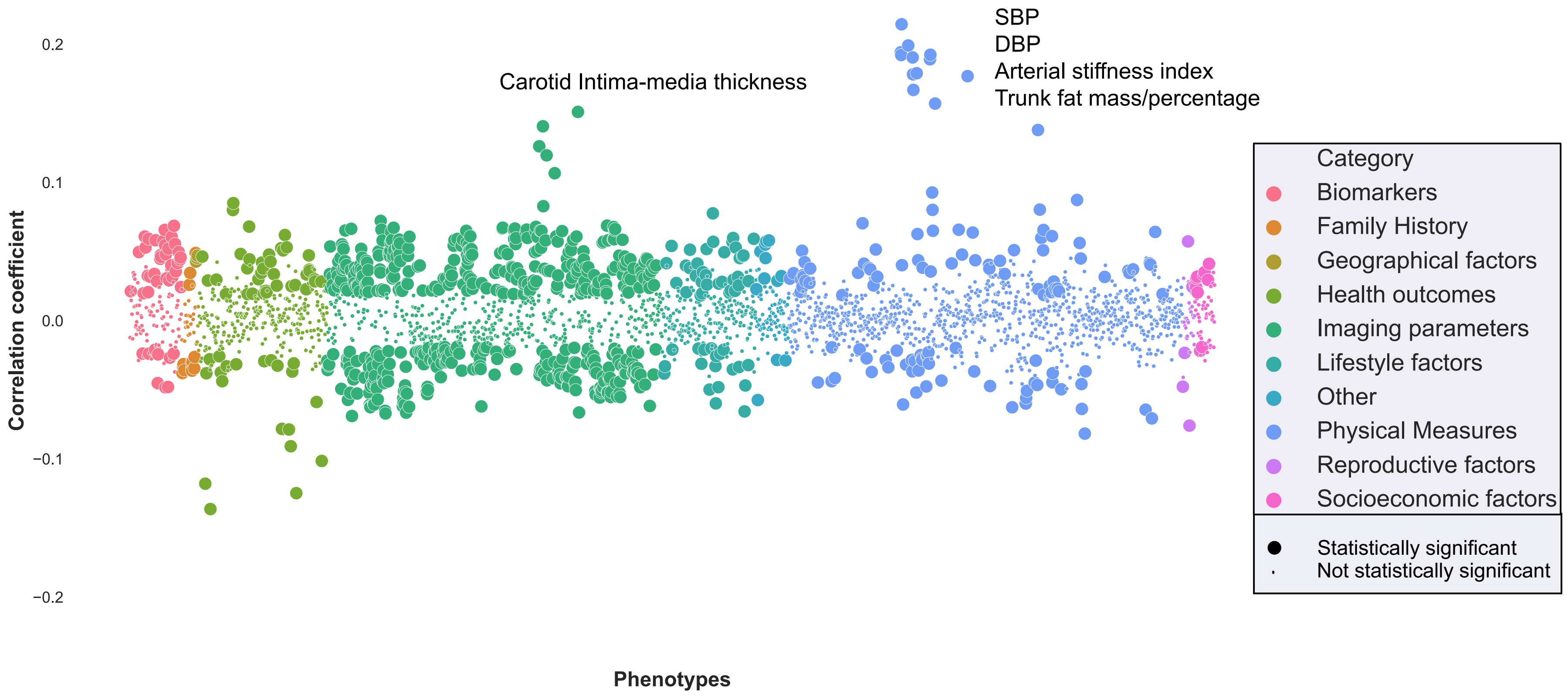

eTable 1
Dataset demographics
Data at the timepoint of a randomly selected ECG per subject is shown for the BIDMC dataset
Categorical variables n (%), continuous variables mean (SD)

	BIDMC	UK Biobank
N subjects	189539	65610
Age	57.68 (18.69)	65.35 (7.88)
Follow up (years)	3.41 (4.08)	3.00 (2.66)
Sex (M)	90792 (47.9)	31825 (48.5)
Hypertension	74409 (39.3)	20673 (31.5)
Previous MI	11788 (6.2)	1610 (2.5)
Smoker	23343 (12.3)	2230 (3.4)
Diabetes Mellitus	33748 (17.8)	3838 (5.8)
Hyperlipidaemia	67087 (35.4)	15866 (24.2)
Mortality	34938 (18.4)	790 (1.2%)

eTable 2
Cohort demographics
Subjects without hypertension at baseline
Categorical variables n (%), continuous variables mean (SD)

	**BIDMC**				
**Risk category**	**Total**	**Low**	**Intermediate-low**	**Intermediate-high**	**High**
**N subjects**	19423	5629	5129	4554	4111
**Age**	50.64 (16.00)	37.82 (12.47)	48.96 (13.06)	56.14 (12.42)	64.19 (12.90)
**Follow up (years)**	6.78 (5.59)	5.90 (5.17)	7.16 (5.72)	7.57 (5.85)	6.61 (5.51)
**Sex (M)**	8260 (42.5)	1632 (29.0)	1948 (38.0)	2227 (48.9)	2453 (59.7)
**Previous MI**	341 (1.8)	17 (0.3)	56 (1.1)	93 (2.0)	175 (4.3)
**Smoker**	1476 (7.6)	296 (5.3)	420 (8.2)	435 (9.6)	325 (7.9)
**Diabetes Mellitus**	1479 (7.6)	245 (4.4)	332 (6.5)	436 (9.6)	466 (11.3)
**Hyperlipidaemia**	4585 (23.6)	782 (13.9)	1332 (26.0)	1380 (30.3)	1091 (26.5)
**Mortality during follow up**	2013 (10.4)	150 (2.7)	325 (6.3)	536 (11.8)	1002 (24.4)
**Hypertension diagnosed during follow up**	6446 (33.2)	753 (13.4)	1456 (28.4)	1960 (43.0)	2277 (55.4)
	**UKB**				
**N subjects**	35806	3789	9584	12999	9434
**Age**	63.51 (7.81)	57.78 (6.50)	61.32 (7.27)	64.16 (7.43)	67.15 (7.28)
**Follow up (years)**	3.96 (2.19)	4.29 (2.08)	4.07 (2.19)	3.93 (2.20)	3.77 (2.21)
**Sex (M)**	15796 (44.1)	1002 (26.4)	3430 (35.8)	6054 (46.6)	5310 (56.3)
**Previous MI**	371 (1.0)	9 (0.2)	34 (0.4)	125 (1.0)	203 (2.2)
**Smoker**	1250 (3.5)	120 (3.2)	380 (4.0)	442 (3.4)	308 (3.3)
**Diabetes Mellitus**	955 (2.7)	33 (0.9)	134 (1.4)	347 (2.7)	441 (4.7)
**Hyperlipidaemia**	5126 (14.3)	237 (6.3)	1026 (10.7)	1895 (14.6)	1968 (20.9)
**Mortality during follow up**	423 (1.2)	25 (0.7)	73 (0.8)	154 (1.2)	171 (1.8)
**Hypertension diagnosed during follow up**	1532 (4.3)	33 (0.9)	189 (2.0)	538 (4.1)	772 (8.2)

eTable 3
Summary table of sex and ethnicity specific AIRE-HTN performance for hypertension prediction in BIDMC Test set

	C-index	95% CI
**Male**	0.680	0.670-0.689
**Female**	0.705	0.696-0.715
**White**	0.698	0.690-0.705
**Black**	0.684	0.666-0.705
**Hispanic**	0.710	0.683-0.744
**Asian**	0.724	0.691-0.754

eTable 4
Summary table of cox model results, comparing AIRE-HTN to clinical risk prediction models in the BIDMC test set
AIRE-HTN-Cox includes AIRE-HTN score, age, sex and ECG parameters.
HTN risk factors include: systolic blood pressure (SBP), diastolic blood pressure (DBP), smoking status, prevalent diabetes mellitus (DM) and ethnicity

	**C-index**	**95% CI**
**ECG parameters**	0.586	0.567-0.604
**Age/Sex**	0.691	0.675-0.707
**AIRE-HTN-Cox**	0.727	0.712-0.742
**Age/Sex/HTN risk factors**	0.734	0.719-0.748
**All except AIRE-HTN**	0.735	0.721-0.750
**All**	0.748	0.734-0.762

eTable 5
Continuous net reclassification index (NRI) (95% confidence interval) for the addition of AIRE-HTN score to a baseline model of age, sex, SBP, DBP, smoking status, prevalent DM, prevalent hypertension, prevalent hyperlipidaemia and ethnicity. UKB analyses additionally included BMI and number of anti-hypertensives as covariates.

Outcome	BIDMC (with or without HTN)	BIDMC (with HTN)	UKB (with or without HTN)	UKB (with HTN)
**CV death**	0.416 (0.250-0.623)	0.194 (0.026-0.444)	0.101 (-0.220-0.398)	0.206 (-0.184-0.616)
**Heart failure**	0.496 (0.413-0.604)	0.338 (0.201-0.450)	0.321 (0.171-0.474)	0.274 (0.043-0.543)
**MI**	0.548 (0.445-0.638)	0.365 (0.214-0.530)	0.206 (0.080-0.306)	0.185 (0.059-0.337)
**Ischaemic stroke**	0.168 (0.059-0.266)	0.110 (-0.011-0.212)	0.194 (0.007-0.380)	0.113 (-0.164-0.291
**Intracranial haemorrhage**	0.268 (0.067-0.478)	0.221 (0.027-0.455)	0.147 (-0.120-0.624)	-0.043 (-0.262-0.765)
**CKD**	0.342 (0.257-0.415)	0.193 (0.081-0.264)	0.124 (-0.055-0.273)	0.085 (-0.092-0.217)

eTable 6
Hypertension related adverse outcomes, number of events in each analysis

Outcome	BIDMC (with or without HTN)	BIDMC (with HTN)	UKB (with or without HTN)	UKB (with HTN)
**N subjects**	32042	14794	48765	13981
**CV death**	325 (1.01%)	174 (1.18%)	128 (0.26%)	64 (0.46%)
**Heart failure**	1154 (3.60%)	615 (4.16%)	351 (0.72%)	182 (1.30%)
**MI**	815 (2.54%)	385 (2.60%)	481 (0.99%)	226 (1.62%)
**Ischaemic stroke**	927 (2.89%)	520 (3.51%)	245 (0.50%)	107 (0.77%)
**Intracranial haemorrhage**	217 (0.68%)	121 (0.82%)	76 (0.16%)	28 (0.20%)
**CKD**	1384 (4.32%)	845 (5.71%)	531 (1.09%)	315 (2.25%)

eTable 7
Mediation analysis
Association of AIRE-HTN score and adverse outcomes, proportion mediated by a diagnosis of hypertension is shown. HF: heart failure, MI: myocardial infarction

	**Proportion mediated**	**Lower 95% CI**	**Upper 95% CI**	**P value**
**BIDMC**				
CV death	21.71%	8.98%	46.59%	<0.0001
MI	11.82%	4.52%	19.71%	<0.0001
HF	5.66%	-3.93%	19.09%	0.36
Haemorrhagic stroke	6.71%	-14.72%	169.88%	0.82
Ischaemic stroke	14.05%	-3.27%	41.58%	0.2
Chronic kidney disease	38.14%	23.66%	66.99%	<0.0001
**BIDMC No HTN at baseline**				
CV death	34.56%	16.85%	167.40%	<0.0001
MI	15.88%	7.39%	25.14%	<0.0001
HF	7.04%	-7.62%	18.11%	0.32
Haemorrhagic stroke	11.46%	-12.84%	70.30%	0.34
Ischaemic stroke	27.22%	11.25%	73.77%	<0.0001
Chronic kidney disease	57.10%	31.26%	110.15%	<0.0001
**UKB**				
CV death	14.77%	8.78%	40.66%	<0.0001
MI	18.97%	8.91%	39.47%	<0.0001
HF	7.46%	1.66%	12.01%	0.02
Haemorrhagic stroke	78.82%	-665.55%	271.87%	0.6
Ischaemic stroke	26.45%	4.54%	120.86%	0.04
Chronic kidney disease	60.11%	-95.78%	1593.73%	0.08
**UKB No HTN at baseline**				
CV death	10.91%	3.86%	30.45%	<0.0001
MI	16.59%	7.48%	79.49%	<0.0001
HF	4.68%	0.88%	10.17%	0.02
Haemorrhagic stroke	359.10%	-624.81%	551.93%	0.76
Ischaemic stroke	22.75%	-77.03%	586.66%	0.08
Chronic kidney disease	72.98%	-437.58%	340.37%	0.58

References

1. Sudlow C, Gallacher J, Allen N, Beral V, Burton P, Danesh J, et al. UK biobank: an open access resource for identifying the causes of a wide range of complex diseases of middle and old age. PLoS Med. 2015;12(3):e1001779.
2. Fry A, Littlejohns TJ, Sudlow C, Doherty N, Adamska L, Sprosen T, et al. Comparison of Sociodemographic and Health-Related Characteristics of UK Biobank Participants With Those of the General Population. Am J Epidemiol. 2017;186(9):1026-34.
3. Bai W, Suzuki H, Huang J, Francis C, Wang S, Tarroni G, et al. A population-based phenome-wide association study of cardiac and aortic structure and function. Nature Medicine. 2020;26(10):1654-62.
4. Ardissino M, McCracken C, Bard A, Antoniades C, Neubauer S, Harvey NC, et al. Pericardial adiposity is independently linked to adverse cardiovascular phenotypes: a CMR study of 42 598 UK Biobank participants. Eur Heart J Cardiovasc Imaging. 2022;23(11):1471-81.
5. Stabenau HF, Waks JW. BRAVEHEART: Open-source software for automated electrocardiographic and vectorcardiographic analysis. Computer Methods and Programs in Biomedicine. 2023;242:107798.
6. Ribeiro AH, Ribeiro MH, Paixao GMM, Oliveira DM, Gomes PR, Canazart JA, et al. Automatic diagnosis of the 12-lead ECG using a deep neural network. Nat Commun. 2020;11(1):1760.
7. Chollet F. Keras 2015 [Available from: https://keras.io.
8. Martín A, Ashish A, Paul B, Eugene B, Zhifeng C, Craig C, et al. TensorFlow: Large-Scale Machine Learning on Heterogeneous Systems. 2015.
9. van de Leur RR, Bos MN, Taha K, Sammani A, Yeung MW, van Duijvenboden S, et al. Improving explainability of deep neural network-based electrocardiogram interpretation using variational auto-encoders European Heart Journal - Digital Health. 2022;3(3):390-404.
10. Stensrud MJ, Hernan MA. Why Test for Proportional Hazards? JAMA. 2020;323(14):1401-2.
11. Whelton PK, Carey RM, Aronow WS, Casey DE, Jr., Collins KJ, Dennison Himmelfarb C, et al. 2017 ACC/AHA/AAPA/ABC/ACPM/AGS/APhA/ASH/ASPC/NMA/PCNA Guideline for the Prevention, Detection, Evaluation, and Management of High Blood Pressure in Adults: Executive Summary: A Report of the American College of Cardiology/American Heart Association Task Force on Clinical Practice Guidelines. Circulation. 2018;138(17):e426-e83.
